# Effectiveness of Incentives for Improving Diabetes-Related Health Indicators in Chronic Disease Lifestyle Modification Programs: a Systematic Review and Meta-Analysis

**DOI:** 10.5888/pcd19.220151

**Published:** 2022-10-27

**Authors:** LaShonda R. Hulbert, Shannon L. Michael, Jasmine Charter-Harris, Charisma Atkins, Renée A. Skeete, Michael J. Cannon

**Affiliations:** 1 Division of Diabetes Translation, National Center for Chronic Disease Prevention and Health Promotion, Centers for Disease Control and Prevention, Atlanta, Georgia; 2 CyberData Technologies, Inc, Herndon, Virginia; 3 Oak Ridge Institute for Science and Education, Oak Ridge, Tennessee; 4 Sapodilla Group, LLC, Atlanta, Georgia

## Abstract

**Introduction:**

We examined the effectiveness of providing incentives to participants in lifestyle modification programs to improve diabetes-related health indicators: body weight, body mass index (BMI), blood pressure, cholesterol, and hemoglobin A_1C_ (HbA_1C_). We also examined the potential effect of 4 different incentive domains (ie, type, monetary value, attainment certainty, and schedule) on those indicators.

**Methods:**

We searched Medline, Embase, PsycINFO, and Cochrane Library to identify relevant studies published from January 2008 through August 2021. We used a random-effects model to pool study results and examine between-study heterogeneity by using the *I* ^2^ statistic and the Cochran Q test. We also conducted moderator analyses by using a mixed-effects model to examine differences between subgroups of incentive domains (eg, incentive type [cash vs other types]).

**Results:**

Our search yielded 10,965 articles, of which 19 randomized controlled trials met our selection criteria. The random-effects model revealed that, relative to the control group, the incentive group had significant reductions in weight (−1.85kg; 95% CI, −2.40 to −1.29; *P* < .001), BMI (−0.47kg/m^2^; 95% CI, −0.71 to −0.22; *P* < .001), and both systolic blood pressure (−2.59 mm HG; 95% CI, −4.98 to −0.20; *P* = .03) and diastolic blood pressure (−2.62 mm Hg; 95% CI, −4.61 to −0.64; *P* = .01). A reduction in cholesterol level was noted but was not significant (−2.81 mg/dL; 95% CI, −8.89 to −3.28; *P* = .37). One study found a significant reduction in hemoglobin A_1c_ (−0.17%; 95% CI, −0.30% to −0.05%; *P* < .05). The moderator analyses showed that the incentive effect did not vary significantly between the subgroups of the incentive domains, except on weight loss for the attainment certainty domain, suggesting that a variety of incentive subgroups could be equally useful.

**Conclusion:**

Providing incentives in lifestyle modification programs is a promising strategy to decrease weight, BMI, and blood pressure.

**Summary**

**What is already known on this topic?**

Lifestyle modification programs can improve diabetes-related health indicators (eg, body mass index, body weight). Including incentives can make these programs more effective.

**What is added by this report?**

We demonstrated the effect of incentives in lifestyle modification programs on multiple diabetes-related health indicators and how this effect might vary by incentive domain (ie, incentive type, monetary value, attainment certainty, and incentive schedule).

**What are the implications for public health practice?**

Using incentives in lifestyle modification programs may improve diabetes-related health indicators, independent of incentive domains. Therefore, programs could exercise flexibility by choosing incentive domains that work for their participants.

**MEDSCAPE CME**

Medscape, LLC is pleased to provide online continuing medical education (CME) for this journal article, allowing clinicians the opportunity to earn CME credit.

In support of improving patient care, this activity has been planned and implemented by Medscape, LLC and *Preventing Chronic Disease*. Medscape, LLC is jointly accredited by the Accreditation Council for Continuing Medical Education (ACCME), the Accreditation Council for Pharmacy Education (ACPE), and the American Nurses Credentialing Center (ANCC), to provide continuing education for the healthcare team.

Medscape, LLC designates this Journal-based CME activity for a maximum of 1.00 *AMA PRA Category 1 Credit(s)™*. Physicians should claim only the credit commensurate with the extent of their participation in the activity.

All other clinicians completing this activity will be issued a certificate of participation. To participate in this journal CME activity: (1) review the learning objectives and author disclosures; (2) study the education content; (3) take the post-test with a 75% minimum passing score and complete the evaluation at http://www.medscape.org/journal/pcd; (4) view/print certificate.

**Release date: October 27, 2022; Expiration date: October 27, 2023**

**Learning Objectives**

Upon completion of this activity, participants will be able to:

Distinguish the most common patient incentive applied in randomized trials included in the current meta-analysis
Assess the effect of patient incentives on body weight and body mass index
Assess the effect of patient incentives on blood pressure
Evaluate other cardiometabolic variables that might be improved with patient incentives

**EDITOR**

Rosemarie Perrin

Editor

Preventing Chronic Disease

Atlanta, Georgia

**CME AUTHOR**

Charles P. Vega, MD

Health Sciences Clinical Professor of Family Medicine

University of California, Irvine School of Medicine

Charles P. Vega, MD, has the following relevant financial relationships:

Consultant or advisor for: GlaxoSmithKline; Johnson & Johnson Pharmaceutical Research & Development, LLC

**AUTHORS**

LaShonda Hulbert, MPH

Centers for Disease Control and Prevention

Atlanta, Georgia

CyberData Technologies, Inc.

Herndon, Virginia

Shannon L. Michael, PhD, MPH

Centers for Disease Control and Prevention

Atlanta, Georgia

Jasmine Charter-Harris, MPH

Centers for Disease Control and Prevention

Atlanta, Georgia

Oak Ridge Institute for Science and Education

Oak Ridge, Tennessee

Charisma Atkins, MPH

Centers for Disease Control and Prevention

Atlanta, Georgia

Renée A. Skeete, PhD

Sapodilla Group, LLC

Atlanta, Georgia

Michael J. Cannon, PhD

Centers for Disease Control and Prevention

Atlanta, Georgia

## Introduction

More than 37 million adults in the US have diabetes, and an additional 96 million have prediabetes, a precursor to type 2 diabetes. Prediabetes is defined as blood glucose levels that are higher than normal, but not high enough to be diagnosed as type 2 diabetes (1). Prevention and management programs are essential for those at risk for and diagnosed with diabetes.

Participating in a lifestyle modification program can help a person develop healthy habits and reduce risks associated with diabetes and related chronic conditions. Results from the National Diabetes Prevention Program have demonstrated that a structured, year-long lifestyle modification program can help participants reduce their risk by coaching them on how to make healthy food choices, reduce stress, and increase physical activity (2,3). Similarly, diabetes self-management education and support services provide essential tools for a person with diabetes to manage the disease and live well (4). However, challenges persist in helping people enroll, stay in, and meet program goals, such as weight loss and blood pressure management (3,5). One promising strategy to address these challenges is the use of incentives (6,7), which can motivate a person to perform a desired action or engage in a behavior (8,9).

Previous systematic reviews and meta-analyses have examined whether incentives can improve health behaviors such as smoking cessation (10,11), getting vaccinations (10), engaging in physical activity (10–16), and improving health indicators such as body weight (11,14). However, some of these reviews did not examine incentives provided solely within the context of a lifestyle modification program (10,11,14,16). Also, several did not examine the effect of different kinds of incentives on program outcomes (10,12,13,15,16).

In contrast, our systematic review and meta-analysis examined a range of diabetes-related health indicators (body weight, body mass index [BMI], blood pressure, cholesterol, and hemoglobin A_1C _[HbA_1c_]) and the effect that incentives might have on them in the context of a lifestyle modification program. We also examined the effect by different types of incentives.

## Methods

We used the PRISMA (Preferred Reporting Items for Systematic Reviews and Meta-Analyses) checklist (17) to guide our systematic review and meta-analysis. At each step in the review process, we worked in pairs to review titles and abstracts for inclusion, extract data, and assess the quality of included studies. We resolved any conflicts through discussion among the authors.

### Data sources

We searched Medline, Embase, PsycINFO, and Cochrane Library databases for peer-reviewed studies published from January 2008 through August 2021.We chose this date range to identify the most up-to-date and culturally and economically relevant information in close proximity to the time period surrounding the US Congressional mandate for the National Diabetes Prevention Program (https://www.congress.gov/bill/111th-congress/house-bill/4124). The search comprised a combination of key terms related to incentives, lifestyle modification programs, and diabetes-related indicators such as weight, BMI, blood pressure, cholesterol, and HbA_1C_ (Appendix).

### Study selection

We selected studies that examined the use of incentives in lifestyle modification programs and their effect on 1 or more diabetes-related health indicators. We considered studies for inclusion if they 1) provided incentives (ie, cash or nonfinancial incentives) to participants; 2) reported on a diabetes-related health indicator(s) (ie, weight, BMI, diastolic and systolic blood pressure, HbA_1C_, or cholesterol); 3) included adults (≥18 y); 4) occurred in high-income countries (18); 5) included a program that incorporated at least 2 of the following components: nutrition, physical activity, and health education; 6) were published in English in a peer-reviewed journal; and 7) were randomized controlled trials (RCTs).

We excluded studies that focused on programs not designed to modify diabetes-related health indicators (eg, weight, blood pressure); medical interventions for weight loss (eg, gastric bypass); pharmaceutical treatment for weight loss; one-time screenings for preventive services; incentives awarded to health care providers or health systems; or conditions or diseases not of interest (eg, infectious diseases, mental disorders, addictions). We excluded all gray literature, conference and dissertation abstracts, and public health presentations.

### Data extraction

We used abstraction forms in DistillerSR (Evidence Partners Incorporated) to screen and manage all articles. Reviewers extracted the following information: study characteristics, study populations, incentive domains, and diabetes-related health indicators. The diabetes-related health indicators of interest were body weight (kilograms [kg] — if only pounds were provided, we converted to kg), body mass index (BMI) (kg/m^2^), blood pressure (both systolic and diastolic in mm Hg), cholesterol (mg/dL), and HbA_1C_ (%). If multiple publications described the same study by using data from the same participants, we selected the publication with the most complete data and excluded the others.

By using a modified version of a previously published framework (19), we extracted data on 6 incentive domains: 1) Type of incentive — the format that was provided to participants. This included cash, noncash financial (incentives that had a monetary value provided in a form other than currency, such as gift cards), nonfinancial (incentives that did not have a specific monetary value, such as water bottles), and mixed (a combination of 2 or more incentive types). 2) Monetary value — the value or worth of incentives provided. Values were categorized as a high amount, defined by the authors as a value of $270 or more, or a low amount, defined as a value less than $270. This value was chosen because it is the median of the maximum amount of money that participants could earn in 17 of the 19 studies included in our review. 3) Recipient — who received the incentive. Recipients could be individuals, a group of individuals, or mixed (a combination of both). 4) Frequency — how often the incentive was provided. Incentives could be provided either once or multiple times throughout the intervention timeframe. 5) Attainment certainty — how certain it was that a recipient would receive an incentive. This included guaranteed certainty, where incentives were provided regardless of criteria being met; criteria-based guaranteed where participants were required to complete an activity or meet a milestone before the incentive was provided; criteria-based lottery, where participants were required to complete an activity, task, or milestone to become eligible for an incentive lottery; lottery, which was an uncertain chance of receiving an incentive that may have been based on completing an activity or meeting a milestone; and mixed, which was a combination of 2 or more of these strategies. 6) Schedule — how the amount of the incentive was provided to recipients during the study period. Incentive schedules included fixed, where participants received the same incentive amount each time no matter what they did or achieved; variable, where recipients received varying incentive amounts over the intervention period; or mixed, which was a combination of both.

We used *The Guide to Community Preventive Services* (20) assessment tool to determine the quality (good, fair, or limited) of each study, summarizing across 6 categories: description, sampling, measurement, analysis, interpretation of results, and other.

### Statistical analysis

For each study arm (ie, incentive group or control group), we extracted the pre- and post- values for diabetes-related health indicators. Using Comprehensive Meta-Analysis Software Version 3 (21), we calculated the effect sizes as the difference between the mean, pre-to-post change in the incentive group and in the control group. When mean pre-to-post changes were not reported, we used other data provided in the study to calculate the mean difference. For studies that used the same control group to compare with multiple incentive groups, we used the mean of the incentive groups in the analyses. A negative difference in means signified that the incentive group lost more weight, had a greater decrease in BMI, or had a greater decrease in blood pressure than the control group.

We used forest plots to compare results of the studies, including differences of means, 95% CIs, and *P* values. We used random effects models, which consider between-study variations, to calculate pooled effect-size estimates. We evaluated the overall effect of studies using *z* statistics with *P* < .05 considered significant. We assessed the risk for publication bias by visually inspecting funnel plots and assessing the degree of asymmetry in the distribution of effect sizes by using the techniques of Begg and Mazumdar (22) and Egger et al (23). When publication bias was detected, we used trim-and-fill procedures to correct for the possibility of missing studies (21).

We assessed heterogeneity by 95% CIs, *I*-squared (*I* ^2^) values, *Q* statistics, and their associated *P* values. We interpreted the *I* ^2^ value, per the Cochrane Handbook (24) as follows: unimportant heterogeneity (0%–40%), moderate heterogeneity (30%–60%), substantial heterogeneity (50%–90%), and considerable heterogeneity (75%–100%). We further assessed heterogeneity by conducting sensitivity analyses that removed individual studies that were potential outliers and assessed the updated findings (ie, forest plots, *I* ^2^ values, and Q statistics). We also examined differences between study quality and types of lifestyle modification programs provided to the incentive and control groups to see how they affected heterogeneity and overall effect size.

To assess the impact of different incentive domains, we conducted moderator analyses with categorical variables by using mixed-effects models. We assessed the effect on body weight and BMI for subgroups within 4 incentive domains: including 1) type (cash vs other types); 2) monetary value (high vs low — we defined high as a value of $270 or more, which is the median of the amount of money that participants could earn in the 17 studies providing financial incentives); 3) attainment certainty (criteria-based guaranteed vs other), and 4) schedule (fixed vs other). Because at least 2 studies in each subgroup are needed to conduct the analysis, we were only able to conduct the moderator analyses for body weight and BMI and for 4 of the 6 incentive domains.

## Results

**Figure 1:**
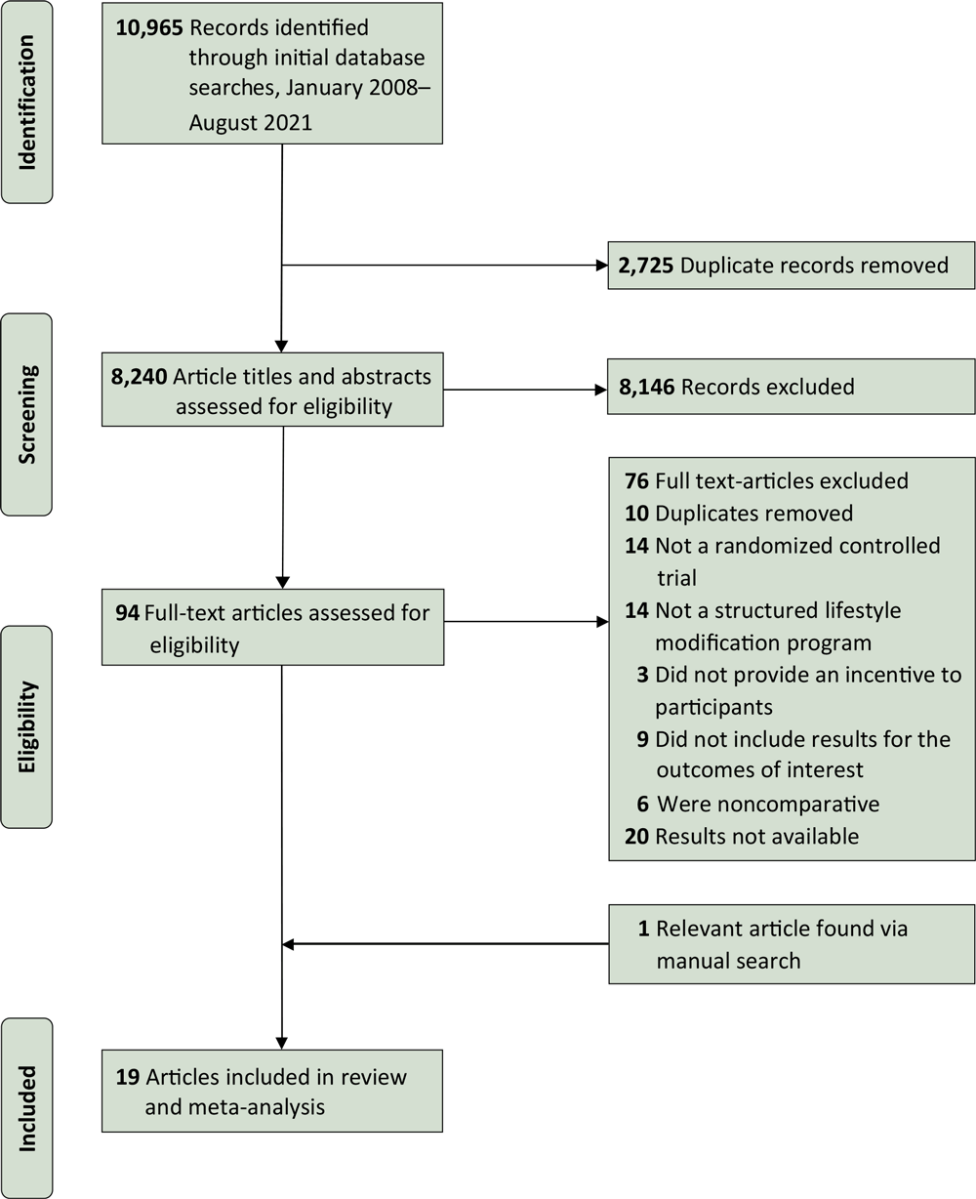
A PRISMA (Preferred Reporting Items for Systematic Reviews and Meta-Analyses) flowchart of the study selection process and literature search from 4 databases: Medline, Embase, PsycINFO, and Cochrane Library, from January 2008 through August 2021. We chose this date range to identify the most up-to-date and culturally and economically relevant information in close proximity to the time period surrounding the US Congressional mandate for the National Diabetes Prevention Program (https://www.congress.gov/bill/111th-congress/house-bill/4124).

Our initial searches returned 10,965 articles (Figure 1). After removing duplicates, we screened 8,240 study titles and abstracts for possible inclusion. On the basis of the inclusion/exclusion criteria, we selected 95 studies for full-text review and identified 19 studies that met the inclusion criteria.

### Study characteristics

Of the 19 studies included in our review, 14 were conducted in the United States, 2 in Australia, 1 in Singapore, 1 in Scotland, and 1 in South Korea (Table 1). Most of the studies had fewer than 500 participants, with a total of 5,291 participants across all studies. The participant age range across studies was 18 to 80 years.

**Table 1:** Characteristics of Study Participants and Programs, Randomized Controlled Trials (N = 19) of Chronic Disease Lifestyle Modification Programs, January 2008–August 2021

First author, year, country	Setting^a^	Population^b^	Number incentive group/nonincentive group	Program description	Intervention duration, final measurement period^c^	Program health indicators of interest	Study quality ratings^d^
Almeida et al, 2015, US (26)	Internet-based, worksite program^e^	Participants aged ≥18 years, BMI ≥25 kg/m^2^, not currently pregnant, free of serious medical conditions (eg, recent heart attack), access to internet	789/1,001	Intervention group received nutrition and physical activity incentives for weight loss and incentives. Comparison group received educational materials focused on nutrition and physical activity without incentives	12 Months; final measurement at month 6	Weight, BMI, cholesterol	Fair
Bennett et al, 2012, US (27)	Community health centers	Participants aged ≥21 years, BMI of 30–50 kg/m^2^, weight <180 kg, and diagnosed hypertension	180/185	Intervention group participated in weight loss activities, engaged with CHWs, and received incentives; comparison group received a self-help booklet and no incentives	24 months, final measurement at month 24	Weight, BMI, systolic and diastolic blood pressure	Fair
Desai et al, 2020, US (30)	Primary care clinics	Participants aged 18–74 years at risk for type 2 diabetes and enrolled in Medicaid	568/279	Intervention group received the 12-month group-delivered DPP, based on the CDC National DPP and incentives. Comparison group received no incentives	12 months, final measurement at month 12	Weight	Fair
Dombrowski et al, 2020, Scotland (31)	Two public health care regions^f^	Men aged ≥18 years, BMI ≥30 kg/m^2^ and/or waist circumference of ≥40 inches, access to a cellular telephone	36/33^g^	Intervention group received narrative text message engagement, physical activity and nutrition components, and incentives. Comparison group received no incentives	12 months, final measurement at month 12	Weight, BMI	Fair
Faghri and Li, 2014, US (32)	Worksite, long-term nursing home facilities	Employees of long-term care facility overweight or with obesity, and at risk for type 2 diabetes	35/38^h^	Intervention group received physical activity and nutrition components and incentives. Comparison group received no incentives	16 weeks, final measurement at week 16	Weight, BMI, systolic and diastolic BP	Fair
Finkelstein et al, 2017, Singapore (33)	Fitness center at Singapore General Hospital	Participants aged 21–65 years; BMI of 25–40 kg/m^2^	107/54	Intervention group received goal setting and tracking, diet and nutrition management, physical activity resources, and incentives. Comparison group received no incentives	8 months, final measurement at month 8	Weight	Fair
John et al, 2011, US (34)	Veterans Affairs Medical Center	Veterans aged 30–70 years, BMI of 30–40 kg/m^2^	A:22, B:22/ control:22	Intervention groups received a weight monitoring program (dietary counseling and weight loss), and 1of 2 incentive plans. Comparison group received no incentives	32 weeks, final measurement at week 32	Weight	Fair
Leahey et al, 2015, US (35)	Internet-based program^e^	Participants aged 18–70 years, BMI ≥25 kg/m^2^	89/91	Intervention group received nutrition, physical activity, weight tracking resources, and incentives. Comparison group received no incentives	3 months, final measurement at month 3	Weight	Good
Leahey et al, 2016, US (29)	Internet-based program^e^	Participants aged 18–70 years, BMI ≥25 kg/m^2^	A:25, B:26/ control:24	Intervention group received coaching, a web-based weight maintenance program based on National DPP, and incentives with either a professional coach or peer coach. Comparison group received a single 1-hour group session and no incentives	12 months, final measurement at month 12	Weight	Fair
Morgan et al, 2011, Australia (25)	Worksite wellness program	Male shift workers at an aluminum company aged 18–65 years who were overweight or had obesity	65/45	Intervention group received a group session on nutrition and physical activity, self-monitoring, goal setting, and incentives. Comparison group was placed on a wait list and did not receive incentives	12 weeks, final measurement at week 14	Weight, BMI, systolic and diastolic blood pressure	Fair
Petry et al, 2011, US (36)	Setting not specified	Participants aged 18–65, BMI of 30–39.9 kg/m^2^, and blood pressure of 110/70–140/90 mm Hg	28/28	Intervention group received nutrition and physical activity components through supportive lifestyle counseling and incentives. Comparison group received no incentives	12 weeks, final measurement at week 12	Weight	Limited
Rounds et al, 2020, US (37)	Internet-based program^e^	Men aged 18–65 years and a BMI of 25–40 kg/m^2^	34/24	Intervention group received nutrition and physical activity components, online lessons, and incentives. Comparison group received no incentives	12 weeks, final measurement at week 24	Weight	Fair
Shin et al, 2017, South Korea (38)	Academic institution	Male students aged 19–45 years, BMI ≥27 kg/m^2^, access to smartphone	35/35^i^	Intervention groups received an individualized education session on nutrition and physical activity and incentives. Comparison group received no incentives	12 weeks, final measurement at week 12	Weight, BMI, systolic and diastolic BP, cholesterol	Fair
Teychenne et al, 2015, Australia (28)	Health and fitness centers or home	Participants aged 40–75 years with type 2 diabetes or BMI ≥25 kg/m^2^	162/156	Intervention group received supervised group exercise sessions, behavioral counseling, newsletters, and incentives. Comparison group received the supervised group exercise sessions and no incentives	12 months, final measurement at month 12	Weight, BMI, HbA_1c_	Limited
VanEpps et al, 2019, US (39)	Community health center, Medicaid managed care plan center, or a local YMCA	Participants aged 18- 64 years, and at risk for type 2 diabetes	170/170^j^	Intervention group received group sessions with physical activity and nutrition components based on the National DPP, and incentives. Comparison group received no incentives	16 weeks, final measurement at week 16	Weight	Good
Voils et al, 2020, US (40)	University medical center	Participants aged 18–70 years, BMI ≥30 kg/m^2^, access to a cellular telephone with a data plan	23/24^k^	Intervention group received the standard behavioral weight loss sessions, nutrition and physical activity components, motivational text messages, and incentives. Comparison group received the standard behavioral weight loss sessions and no incentives	24 weeks, final measurement at week 24	Weight	Fair
Volpp et al, 2008, US (41)	Veterans Affairs medical center	Participants aged 30–70 years, BMI of 30–40 kg/m^2^	A:19, B:19/ control:19	Intervention group received an individual session with a dietician, physical activity, nutrition components, and 1 of 2 incentive plans. Comparison group received no incentives	16 weeks, final measurement at week 16	Weight	Good
West et al, 2020, US (42)	Internet-based program^e^	Participants aged ≥18 years, BMI of 25–50 kg/m^2^, and access to the internet	206/212	Intervention group received online group-based behavioral weight control sessions based on the National DPP, with physical activity and nutrition components and incentives. Comparison group received no incentives	6 months, final measurement at month 6	Weight	Fair
Yancy et al, 2018, US (43)	Internet-based program^e^	Participants enrolled in Weight Watchers aged 30–80 years, BMI of 30–45 kg/m^2^	A:75, B:77/ control: 39	Intervention group received physical activity and nutrition components, text message engagement, and was assigned to 1 of 2 incentive plans. Comparison group received no incentives	6 months, final measurement at month 6	Weight	Good

Abbreviations: BP, blood pressure; BMI, body mass index; CDC, Centers for Disease Control and Prevention, CHW, community health worker; National DPP, National Diabetes Prevention Program; YMCA, Young Men’s Christian Association.

a The location where the study took place or the location of the principal investigators where participants would report.

b Because age, BMI, and gender (if focused only on males or females) were consistently reported in the included studies, we were able to include this demographic information across all studies for the populations of focus. Other demographic information such as race, was not consistently reported across the studies and we therefore did not include it here.

c The length of time that participants receive program support. We defined final measurement period as the last point at which health indicators were measured and the incentive groups were still receiving support — this is the health indicator measure used for our meta-analysis.

d Consists of 3 categories — good, fair, and limited — measuring across 6 categories: description, sampling, measurement, analysis, interpretation of results, and other. These ratings are explained in the *Guide to Community Preventive Services *assessment tool (15).

e Defined as a lifestyle modification program that is delivered via the internet, including access to program materials and engagement with program facilitators and peers if applicable.

f Scotland is divided into health care regions in which the public receives health care services.

g This study had 3 arms. For the purposes of our analysis, we compared the arm that received the intervention plus incentives to the arm that received only the intervention versus the wait-list control arm.

h This study had 3 arms. For the purposes of our analysis, we compared the collective arm that combined both incentive groups to the control group.

i This study had 3 arms. For the purposes of our analysis, we compared the arm that received the intervention plus incentives to the control group.

j This study had 4 arms. For the purposes of our analysis, we compared the arm that received a combination of incentives to the control group.

k This study had 4 arms. For the purposes of our analysis, we compared the arm that received incentives for weight loss and self-monitoring to the control group.

The most frequently reported setting of the included studies was internet-based programs (n = 6) (26,29,35,37,42,43). The time frame for the lifestyle modification programs was from 12 weeks to 24 months. All included studies measured weight, 7 measured BMI (25–28,31,32,38), 4 measured blood pressure (25,27,32,38), 2 measured cholesterol (26,38), and 1 measured hemoglobin A_1c_ (HbA_1c_) (28). The quality scores of the studies varied: 4 good (35,39,41,43); 13 fair (25–27,29–34,37,38,40,42); and 2 (28,36) limited.

### Incentive domains

Among the 19 RCTs, the most common incentive type was cash (Table 2). Most studies reported a monetary value greater than or equal to the median of $270 (n = 9) (30–34,37,38,40,43), and most incentives were distributed to individual recipients (n = 15) (26–29,31–33,35–40,42,43). Incentives were frequently provided multiple times over the course of the included programs (n = 16) (25–30,33,34,36–43); most studies used a criteria-based guaranteed approach (n = 15) (25,26,29–34,37–43), and 13 studies (26,27,29–31,33–38,41,42) distributed the incentive to participants on a variable schedule.

**Table 2:** Summary of Incentive Domain Characteristics, Randomized Controlled Trials (N = 19) of Chronic Disease Lifestyle Modification Programs, January 2008–August 2021

Author, year	Incentive description	Incentive type^a^	Monetary value^b^	Recipient^c^	Frequency^d^	Attainment certainty^e^	Schedule^f^
Almeida et al, 2015, (26)	Participants could receive cash based on percentage weight loss at quarterly weigh-ins (eg, 1% weight loss = $1.00, 2% weight loss = $2.00). Maximum potential earnings, ~$5 USD	Cash	Low	Individual	Multiple	Criteria-based guaranteed	Variable
Bennett et al, 2012 (27)	Participants could receive a grocery card ($50 USD) at completion of baseline, 6-, 12-, and 18-month visits and a grocery card ($75 USD) at 24 months. Participants also received a scale at 12-month visit and a blood pressure monitor at 18-month visit. Maximum potential earnings, $125 USD	Mixed	Low	Individual	Multiple	Guaranteed	Variable
Desai et al, 2020 (30)	Participants could receive incentives via a reloadable debit card for attendance and weight loss goals over the 12 months. Maximum potential earnings, $520 USD	Noncash financial	High	Mixed	Multiple	Criteria-Based Guaranteed	Variable
Dombrowski et al, 2020 (31)	At baseline, researchers deposited £400 GBP (~$550 USD) in a hypothetical bank account, and participants could secure/lose certain amounts when specific targets were reached/not reached. Maximum potential earnings, £400 GBP (~$550 USD)	Cash	High	Individual	Once	Criteria-Based Guaranteed	Variable
Faghri et al, 2014 (32)	Participants could receive cash for every 1 to 1.5 lb lost. Participants could choose 1 of 2 incentive plan: simple financial reward of $10 per lb of weight loss (maximum earnings, $260 USD) or simple financial reward plus deposit where participants could deposit $1–$5 per lb. of weight loss including a 1:1 match from the researchers. Maximum potential earnings, $340 USD	Cash	High	Individual	Once	Criteria-based guaranteed	Mixed
Finkelstein et al, 2017 (33)	Participants first paid a refundable fee to participate in the incentive plan. Participants could receive guaranteed cash payments or lottery cash prizes. Maximum potential earnings, S$600 SGD ($488 USD)	Cash	High	Individual	Multiple	Criteria-based guaranteed	Variable
John et al, 2011 (34)	Participants deposited their own money ($1–$3 per day) into a hypothetical account with a 1:1 match from the researchers. Incentive group A had a weight maintenance period weeks 25–32 and incentive group B did not. Maximum potential net earnings, $672 USD	Cash	High	Mixed	Multiple	Criteria-based guaranteed	Variable
Leahey et al, 2016 (29)	Participants could receive cash payments ($1–$10) weekly for submitting self-monitoring records and diet or activity information. An additional $25 was provided for maintaining weight loss. Maximum potential earnings, $185 USD	Cash	Low	Individual	Multiple	Criteria-based guaranteed	Variable
Leahey et al, 2015 (35)	Participants could receive cash payments ($1–$10) for submitting weight, nutrition, and activity information to be distributed after their 3-month assessment. Maximum potential earnings, $45 USD	Cash	Low	Individual	Once	Mixed	Variable
Morgan et al, 2011 (25)	Participants could receive sporting store gift vouchers (AU$50 [$37 USD]) per crew member based on the group that achieved the greatest mean weight loss and program completion. Maximum value, AU$100 (~$73 USD)	Noncash financial	Low	Group	Multiple	Criteria-based guaranteed	Fixed
Petry et al, 2011 (36)	Participants drew from a bowl with a chance to receive small incentives (healthy snacks, bottled water, toiletries) or large incentives ($20 gift cards, weight sets) worth $1–$100 USD for weight loss and completing weight loss activities.	Mixed	NA	Individual	Multiple	Criteria based lottery	Variable
Rounds et al, 2020 (37)	Participants could receive escalating incentives weekly for weight loss with a reset contingency if weekly weight loss goals were not met, starting at $4 USD in the first week. Maximum potential Earnings, $312 USD	Cash	High	Individual	Multiple	Criteria-based guaranteed	Variable
Shin et al, 2017 (38)	Participants could receive incentives for meeting daily physical activity goals and weight loss goals. Maximum potential earnings, ₩ 320,000 KRW (~$270 USD)	Cash	High	Individual	Multiple	Criteria-based guaranteed	Variable
Teychenne et al, 2015 (28)	Participants could receive motivational incentives such as a sports bag or water bottle.	Nonfinancial	NA	Individual	Multiple	Guaranteed	Fixed
VanEpps et al, 2019 (39)	Participants could receive incentives for attending group sessions and meeting weight loss goals. Maximum potential earnings, $240 USD	Cash	Low	Individual	Multiple	Criteria-based guaranteed	Fixed
Voils et al, 2020 (40)	Participants could receive incentives weekly (up to $30 USD per week on a reloadable debit card) for dietary self-monitoring and weight loss. Maximum potential earnings, $300 USD	Noncash financial	High	Individual	Multiple	Criteria-based guaranteed	Mixed
Volpp et al, 2008 (41)	Participants could receive incentives through a deposit contract or lottery incentive plan for meeting weight loss goals. The deposit contract required participants to deposit their own money ($1–$3) daily, which was matched 1:1 with an extra fixed payment of $3 per day and was refundable upon meeting or exceeding weight loss goals. The lottery plan offered a chance to receive daily incentives with a value of $3. Maximum net potential earnings, $168 USD	Cash	Low	Mixed	Multiple	Criteria-based guaranteed	Variable
West et al, 2020 (42)	Participants could receive incentives ($10–$15 per week via Amazon gift card) for submitting diet records and meeting weight loss goals. Maximum potential earnings, $230 USD	Noncash financial	Low	Individual	Multiple	Criteria-based guaranteed	Variable
Yancy et al, 2018 (43)	Participants could receive incentives through direct payments or lottery incentive plans for meeting weight loss goals. Maximum potential earnings, ~$590 USD	Cash	High	Individual	Multiple	Criteria-based guaranteed	Mixed

Abbreviations: NA, not applicable; USD, US dollar; GBP, British pound sterling; SGD, Singapore dollar; AU, Australian dollar; KRW, South Korean won.

a The format of the incentive. Cash incentives were provided to participants in $USD currency or the currency of the study country’s location. Noncash financial incentives had a monetary value in a form other than currency (eg, gift cards, childcare or dependent assistance, transportation or store vouchers, health care premium discounts). Nonfinancial incentives were in kind and did not have a specific monetary value (eg, fitness equipment, products, various prizes). Mixed incentives were a combination of 2 or more incentive types.

b The worth of incentives provided to recipients for their participation in the program. A high amount, as defined by the authors, had a value of $270 or more. A low amount was defined as a value less than $270. This value was chosen because it is the median amount of money that participants could earn in 17 of the 19 studies included in our review.

c Who received the incentive: individuals, a group of individuals, or mixed (a combination of both).

d How often the incentive was provided to the recipient. Incentives could be given either once or multiple times throughout the intervention timeframe.

e How certain it was that a recipient would receive an incentive. Guaranteed = provided regardless of criteria being met; criteria-based guaranteed = must complete an activity or meet a milestone before the incentive is provided; criteria-based lottery = must complete an activity, task, or milestone to become eligible for an incentive lottery; lottery = an uncertain chance of receiving an incentive that could be based on completing an activity or meeting a milestone; mixed = a combination of 2 or more of these strategies.

f How the amount of the incentive was provided to recipients. Rates were based on what the recipient could potentially receive and were usually contingent on an activity, task, or timing. Fixed = the same incentive amount was given to recipients each time no matter what they did or achieved; variable = a varying incentive amount was provided to recipients over the intervention period; mixed = a combination of both.

### Magnitude and direction of effects

**Figure 2:**
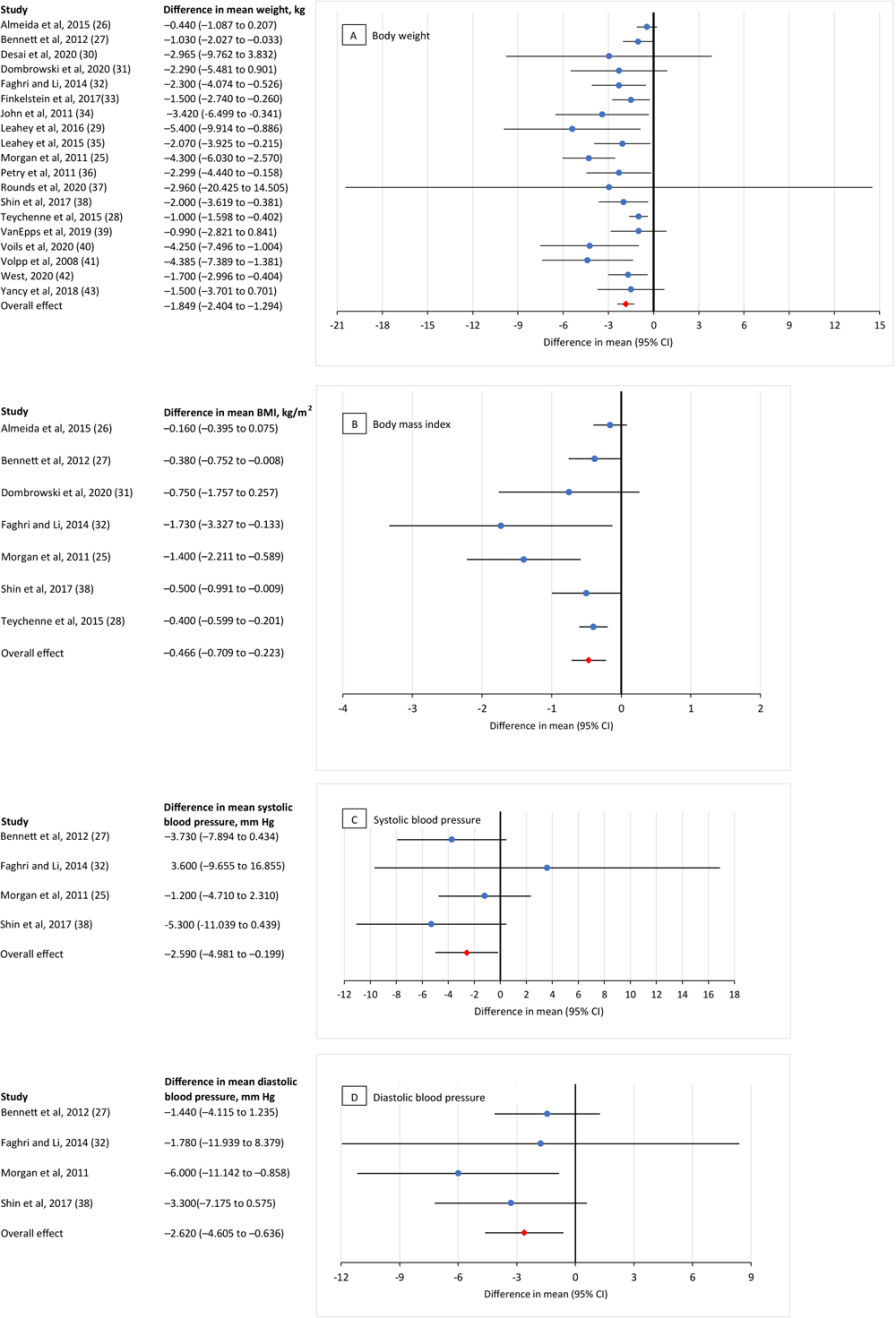
Meta-analysis of the effects of incentives (ie, cash or nonfinancial incentives) on improving diabetes-related health indicators in chronic disease lifestyle modification programs. A, the effect of incentives on body weight (kg); calculations were based on 23 comparisons reported in 19 studies (25–43). B, the effect of incentives on body mass index (kg/m^2^); calculations were based on 7 comparisons reported in 7 studies (25–28,31,32,38). C, the effect of incentives on systolic blood pressure (mm Hg); calculations were based on 4 comparisons reported in 4 studies (25,27,32,38). D, the effect of incentives on diastolic blood pressure (mm Hg); calculations were based on 4 comparisons reported in 4 studies (25,27,32,38). Values <0 indicate an incentive effect, and values >0 indicate no incentive effect.

We found a significant overall mean effect for providing incentives in lifestyle modification programs for 4 diabetes-related health indicators: body weight, BMI, and both diastolic and systolic blood pressure (Figure 2).

**Table:** 

Study	Difference in means (BMI in kg/m2)	Lower limit	Upper limit
A. Effect of incentives on body weight, kg			
Almeida et al, 2015 (26)	–0.440	–1.087	0.207
Bennett et al, 2012 (27)	–1.030	–2.027	–0.033
Desai et al., 2020 (30)	–2.965	–9.762	3.832
Dombrowski et al, 2020 (31)	–2.290	–5.481	0.901
Faghri and Li, 2014 (32)	–2.300	–4.074	–0.526
Finkelstein et al, 2017 (33)	–1.500	–2.740	–0.260
John et al., 2011 (34)	–3.420	–6.499	–0.341
Leahey et al, 2016 (29)	−5.400	–9.914	–0.886
Leahey et al, 2015 (35)	–2.070	–3.925	–0.215
Morgan et al, 2011 (25)	–4.300	–6.030	–2.570
Petry et al, 2011 (36)	–2.299	–4.440	–0.158
Rounds et al, 2020 (37)	–2.960	–20.425	14.505
Shin et al, 2017 (38)	–2.000	–3.619	–0.381
Teychenne et al, 2015 (28)	–1.000	–1.598	–0.402
VanEpps et al, 2019 (39)	–0.990	–2.821	0.841
Voils et al, 2020(40)	–4.250	–7.496	–1.004
Volpp et al, 2008 (41)	–4.385	–7.389	–1.381
West, 2020 (42)	–1.700	–2.996	–0.404
Yancy et al, 2018 (43)	–1.500	–3.701	0.701
Overall effect	–1.849	–2.404	–1.294
B. Effect of incentives on body mass index (kg/m2),			
Almeida et al, 2015 (26)	–0.160	–0.395	0.075
Bennett et al, 2012 (27)	–0.380	–0.752	–0.008
Dombrowski et al, 2020 (31)	–0.750	–1.757	0.257
Faghri and Li, 2014 (32)	–1.730	–3.327	–0.133
Morgan et al, 2011 (25)	–1.400	–2.211	–0.589
Shin et al, 2017 (38)	–0.500	–0.991	–0.009
Teychenne et al, 2015 (28)	–0.400	–0.599	–0.201
Overall effect	–0.466	–0.709	–0.223
C. Effect of incentives on systolic blood pressure (mm Hg)			
Bennett et al, 2012 (27)	–3.730	–7.894	0.434
Faghri and Li, 2014 (32)	3.600	–9.655	16.855
Morgan et al, 2011 (25)	–1.200	–4.710	2.310
Shin et al, 2017 (38)	–5.300	–11.039	0.439
Overall effect	–2.590	–4.981	–0.199
D. Effect of incentives on diastolic blood pressure, mm Hg			
Bennett et al, 2012 (27)	–1.440	–4.115	1.235
Faghri and Li, 2014 (32)	–1.780	–11.939	8.379
Morgan et al, 2011 (25)	–6.000	–11.142	–0.858
Shin et al, 2017 (38)	–3.300	–7.175	0.575
Overall effect	–2.620	–4.605	–0.636

**Weight.** The pooled mean difference for weight was −1.85 kg (95% CI, −2.40 to −1.29; *Z* = −6.53, *P* < .001), indicating that the incentive group lost more weight (−1.85 kg or 4.1lb) than the control group.

**BMI**. The pooled mean difference for BMI was −0.47 kg/m^2^ (95% CI, −0.71 to −0.22; Z = −3.76, *P* < .001), meaning the incentive group decreased their BMI by 0.47 kg/m^2^ more than the control group.

**Blood pressure.** The pooled mean difference for systolic blood pressure was −2.59 mm Hg (95% CI, −4.98 to −0.20; Z = −2.12, *P* = .03), and for diastolic blood pressure it was −2.62 mm Hg (95% CI, −4.61 to −0.64; Z = −2.59, *P* = .01) meaning that the incentive group had a greater decrease in their systolic and diastolic blood pressure than the control group.

**Cholesterol and HbA_1C_**. Two studies (26,38) examined total cholesterol as an outcome measure and both were nonsignificant, with a pooled mean difference of −2.81 mg/dL (95% CI, −8.89 to −3.28; Z = −0.91, *P* = .37). We did not have at least 2 studies to conduct a meta-analysis for HbA_1C_; in the only RCT (28) examining that value, the incentive group had a significantly greater decrease in their HbA_1C_ levels than the control group (mean difference, −0.17%; 95% CI, −0.30 to −0.05, *P* = <.05).

### Publication bias

The funnel plots of the effect sizes for weight and BMI were asymmetrical, suggesting possible publication bias for studies with nonsignificant effects. This was supported by the significant findings from the Begg and Mazumdar (22) publication bias test results for weight (Kendall's tau b = −0.33, with a one-tailed *P* value of .03) and BMI (Kendall's tau b = −0.57, with a one-tailed *P *value of .04). In addition, the Egger et al (23) publication bias test showed an intercept of −1.78 with a one-tailed *P* value of <.001 for weight, and an intercept of −1.93 with a one-tailed *P* value of .02 for BMI. We used Duval and Tweedie’s Trim and Fill (21) analysis to understand this further and found that 9 potential studies might be missing from the weight meta-analysis because of publication bias. With these additional studies, the estimated effect size for weight would be smaller, −1.20 kg, but still significant (95% CI, −1.80 to −0.60). For BMI, we found that 3 potential studies might be missing from the BMI meta-analysis, and with these additional studies, the estimated effect size for BMI would be smaller, −0.34 kg/m^2^, but still significant (95% CI, −0.62 to −0.06).

The funnel plots of the effect sizes were symmetrical for diastolic and systolic blood pressure, and the trim-and-fill analysis suggested no adjustment to the mean effect size, indicating no evidence of publication bias.

### Heterogeneity and subgroup analyses

We found moderate to substantial heterogeneity for the effect size of incentives on weight (*I* ^2^ = 51%; *Q* = 36.43; *P* < .01) and BMI (*I* ^2^ = 54%; *Q* = 12.95; *P* = .04), so we conducted subgroup analyses to better understand the potential sources of heterogeneity.

First, we identified potential study outliers as those with standard residuals ≥1.96. One study was identified as an outlier for weight and BMI (25). When we removed this study as a potential outlier, the *I* ^2^ percentage decreased to nonsignificant heterogeneity (*I* ^2^ = 31%, *Q* = 24.77, *P* = .10) for weight and for BMI (*I* ^2^ = 24%, *Q* = 6.60, *P* = .25). We chose to keep this study in both meta-analyses because it did not change the significance and only slightly lowered the pooled effect sizes (weight: from −1.85 kg to −1.54 kg; BMI: from −0.47 kg/m^2^ to −0.36 kg/m^2^).

Second, we assessed heterogeneity as a function of study quality but did not find a consistent pattern. We found that for weight, studies rated as fair (25–27,29,30–34,37,38,40,42) had a stronger effect size (−2.07 kg) and moderate to substantial heterogeneity (*I* ^2^ = 60%) compared with studies rated as good (35,39,41,43) (−1.92 kg; *I* ^2^ = 20%, unimportant heterogeneity) and limited (28,36) (−1.23 kg; *I* ^2^ = 24%, unimportant heterogeneity). For BMI, the assessment was constrained because 6 studies were rated as fair (25–27,31,32,38) but only one was rated limited (28). The fair studies had a stronger effect size (−0.56 kg/m^2^) and moderate to substantial heterogeneity (*I* ^2^ = 61%) compared with the study rated as limited (−0.40kg/m^2^; *I* ^2^ = 0%, unimportant heterogeneity).

Lastly, we assessed heterogeneity as a function of program characteristics. For weight, there were 5 studies that provided slightly different lifestyle modification programs to the incentive group and the control group (25–29), whereas 14 studies provided the same program to both groups, with the incentive being the only difference (30–43). We found that the effect size was greater for the 14 studies that provided the same program to the incentive and control groups (−1.99 kg) compared with the 5 studies that did not (−1.62 kg). In addition, for those 14 studies the *I* ^2^ was 0% and nonsignificant (*Q* = 7.59; *P* = .87), which means that the differences across those studies are due to sampling error, not differences in true effect sizes. For the 5 studies that did not provide the same program, the *I* ^2^ was 80% and significant (*Q* = 20.51; *P* = <.001), which means the amount of variability across the studies cannot be explained by chance alone. We found similar results for BMI, where the three studies (31,32,38) that provided the same program to the incentive group and control group had a greater effect size (−0.65 kg/m^2^) and unimportant heterogeneity (*I* ^2^ = 6%; *Q* = 2.15; *P* = .34), compared with the lower effect size (−0.41 kg/m^2^) with substantial heterogeneity (*I* ^2^ = 67%; *Q* = 9.20; *P* = .03) for the 4 studies (25–28) that provided a slightly different program to both groups.

We found heterogeneity to be unimportant for diastolic (*I* ^2^ = 0%, *Q* = 2.55; *P* = .47) and systolic (*I* ^2^ = 0%, *Q* = 2.58, *P* = .46) blood pressure, so we did not conduct any further analyses.

### Moderator analyses for incentive domains

The effect sizes between most of the subgroups of the incentive domains were not significantly different (Table 3), indicating no difference between each subgroup’s ability to lower body weight and BMI. For the attainment certainty domain, both subgroups had an effect on weight, but the difference between them was significant, indicating that the criteria-based guaranteed subgroup might have a greater effect on weight (−2.20 kg) than other attainment criteria (−1.15 kg).

**Table 3:** Moderator Analysis of Incentive Domain Subgroups by Diabetes-Related Health Indicators, Randomized Controlled Trials (N = 19) of Chronic Disease Lifestyle Modification Programs, January 2008–August 2021

Incentive domain and subgroup	N (mean difference) [95% CI]	*P* Value^a^	I^2 ^(%)	Difference between subgroups^b^
**Weight**				
Type^c^				
Cash	12 (−1.79) [−2.53 to −1.05]	<.001	42.14	*Q* = 0.15, *P* = .69
Other types	7 (−2.03) [−3.01 to −1.06]	<.001	64.85	
Monetary value^d^				
High	9 (−2.04) [−2.77 to −1.32]	<.001	0.00	*Q* = 0.00, *P* = .95
Low	8 (−2.01) [−3.05 to −0.96]	<.001	73.93	
Attainment certainty^e^				
Criteria-based guaranteed	15 (−2.20) [−3.01 to −1.40]	<.001	57.59	*Q* = 4.92, *P* = .03
Other	4 (−1.15) [−1.63 to −0.66]	<.001	0.00	
Schedule^f^				
Fixed	3 (−2.02) [−4.00 to −0.03]	.046	84.15	*Q* = 0.04, *P* = .83
Other schedules	16 (−1.80) [−2.39 to −1.20]	<.001	34.97	
**Body mass index (weight in kg divided by height in m^2^)**				
Type^c^				
Cash	4 (−0.44) [−0.86 to −0.03]	.034	47.98	*Q* = 0.14, *P* = .71
Other types	3 (−0.55) [−0.93 to −0.17]	.005	64.51	
Monetary Value^d^				
High	3 (−0.65) [−1.12 to −0.19]	.010	6.81	*Q* = 0.20, *P* = .65
Low	3 (−0.50) [−0.99 to −0.00]	.048	76.73	
Attainment certainty^e^				
Criteria-based guaranteed	5 −0.69 [−1.21 to −0.18]	.008	68.67	*Q* = 1.15, *P* = .28
Other	2 (−0.39) [−0.57 to −0.22]	<.001	0.00	
Schedule^f^				
Fixed	2 (−0.82) [−1.79 to −0.15]	.097	81.84	*Q* = 0.77, *P* = .38
Other schedules	5 (−0.37) [−0.64 to −0.10]	.007	33.58	

Abbreviations: *Q*, Cochrane *Q* statistic.

a Refers to whether the association between the incentive domain subgroup and the health indicator was significant, based on the z value in the mixed-effects analysis.

b Refers to whether the mean differences for the 2 subgroups were statistically different from each other, based on the Q value in the mixed-effects analysis.

c Cash is currency provided to participants; other types include noncash financial, nonfinancial, or mixed.

d High includes studies that provided incentives valued at $270 or more; low includes studies that provided incentives valued at less than $270. For weight, the Petry et al (36) and Teychenne et al (28) studies were not included in the moderator analysis because they did not have a cash value. For BMI, the Teychenne et al (28) study was not included because it did not have a cash value.

e Criteria-based guaranteed includes studies where an activity, task, or milestone must have been met before incentives were provided; other includes guaranteed (incentive provided regardless of criteria being met), criteria-based lottery (an activity, task, or milestone must be completed to be eligible for an incentive lottery), and mixed (a combination of 2 or more of these strategies).

f Fixed means the same incentive amount was given to recipients each time, no matter what they did or achieved; other schedules include variable (a varying incentive amount was given to recipients over the intervention period) and mixed (a combination of both schedules).

## Discussion

To our knowledge, this is the first systematic review and meta-analysis to examine the effect of incentives on multiple diabetes-related health indicators in the context of a lifestyle modification program. Our findings suggest that incentives are an effective strategy to lower body weight, BMI, and systolic and diastolic blood pressure. Of note, the results for HbA_1c_ were significant, but only 1 study reported on this (28). Although the results for cholesterol were promising, they were not significant. The results of the subgroup analyses showed that in programs where the only difference between incentive and control groups was the incentive, a larger effect on body weight and BMI was observed, which is indicative of the usefulness of incentives and the reliability of the effect size.

The results of our meta-analysis showed a nearly 2 kg greater weight loss and a significant reduction in BMI when incentives were provided. This finding has important health implications considering that a large study reported a 16% reduction in diabetes risk for every kg of weight lost (44), and others have shown that weight loss in conjunction with a lifestyle modification program can lower the risk for cardiovascular disease (45–47). Additionally, for those with type 2 diabetes who are overweight or have obesity, there are significant benefits for reducing not only body weight, but blood pressure, and cholesterol as well (45).

Providing incentives to participants in lifestyle modification programs was shown to be effective for reducing systolic and diastolic blood pressure. These findings could be beneficial for programs that seek to help participants achieve the ideal blood pressure (ie, <140/90 mm Hg), especially those programs associated with the prevention and management of chronic diseases such as type 2 diabetes (48). Reducing blood pressure protects against cardiovascular events for people at risk for and diagnosed with diabetes and is critical to managing the disease (48,49).

The results of the moderator analyses showed that nearly all the subgroups in each incentive domain (eg, high vs low monetary value) had a significant effect on reducing body weight and BMI. Some of the effect sizes were larger than others, suggesting that certain incentive domains might be more effective. For example, for the incentive type domain, the cash subgroup showed a −1.79 kg (*P* = <.001) decrease in weight, whereas the other subgroup types showed a −2.03 kg (*P* = <.001) decrease. However, the differences in effect sizes across most subgroups were mostly nonsignificant. Therefore, it seems reasonable for lifestyle modification programs to use a variety of incentive domain subgroups.

Future studies could consider the effectiveness of various types of incentives for specific populations, cultures, certain health indicators like cholesterol and HbA_1c_, and certain settings to determine whether incentives could reduce disparities in the outcomes of lifestyle modification programs (50,51). Although recipients of incentives generally find them acceptable (52–55), other individual and programmatic considerations, such as demographic characteristics or funding sources, deserve further exploration (53). Similarly, researchers could consider using a common framework (10–12,19) for reporting incentives in addition to reporting program and participant characteristics so that they might be examined simultaneously. Also, evaluating incentives for cost-effectiveness could ensure that resources spent on them make economic sense for programs. Future research might also seek to better understand how using incentives might affect intrinsic motivation or habit formation, especially because lifestyle modification requires a long-term commitment to the habitual behaviors that reduce risks (56–58). One study has reported that it is unlikely that incentives undermine intrinsic motivation and could possibly enhance intrinsic motivation to participate in a program (59).

### Limitations

Our study has several limitations. Variability across lifestyle modification programs and incentive domains likely contributed to the heterogeneity of our results. Nevertheless, we found no significant differences in effect sizes according to the subgroup analysis for heterogeneity. Also, 2 of the incentive subgroup moderator analyses consisted of 2 studies, so we interpreted those results with caution. Finally, we were not able to include other factors that could influence participation or success, such as race or ethnicity of participants or the coach, the socioeconomic status of participants, or other incentive domains, such as incentive timing (19), so our results are limited to what we could abstract from the existing studies.

### Strengths

Our study has several strengths. We had enough RCT studies to include in a meta-analysis with multiple diabetes-related health indicators, and most of them were considered of good or fair quality, mostly on the basis of limitations of the sample designs and the interpretation of study results. We used subgroup analyses to address heterogeneity, used moderator analyses to understand the effect of several incentive domains on health indicators, and adjusted for potential publication bias. Through these analyses we determined that the observed effect of the incentives could not be attributed to outlier studies or publication bias and that the effect sizes were consistent across incentive domains. Furthermore, for the programs that differed only by the incentive, we observed a strong effect and low heterogeneity in these studies for body weight and BMI. These findings provide more confidence that the use of incentives in lifestyle modification programs for chronic disease can lead to better outcomes.

### Conclusion

Using incentives in lifestyle modification programs is a promising strategy for adults at risk for or diagnosed with type 2 diabetes to reduce body weight, BMI, blood pressure, and, potentially, HbA_1c_. Because our analysis showed effectiveness of incentives on multiple health indicators, we were able to add to the literature regarding the use of incentives to promote lifestyle modification. Chronic disease prevention and management programs can consider incentives as a tool to increase participant success.

## Appendix

**Table:** Systematic Review Search Strategy, Randomized Controlled Trials of Chronic Disease Lifestyle Modification Programs, January 2008–August 2021^a^

Database	Strategy
Medline (OVID) 1946–	(money OR cash OR incentiv* OR token econom* OR token reinforcement* OR payment* OR paid OR earn* OR reimburse* OR wage* OR contingency management OR coupon* OR discount OR voucher* OR gift* OR free food OR prize* OR award* OR reward*).mp. AND (Weight reduction OR weight management OR weight loss* OR obesity OR overweight OR body weight OR body mass OR bmi OR excess weight OR diet* OR feeding behavior OR eating OR nutrition OR lifestyle OR life style OR behavio?r change OR behavior modification OR behavior therapy OR health promotion OR health behavior OR healthy OR fitness OR exercise OR exercising OR physical activit* OR strength training OR diabetes OR diabetic OR prediabet* OR pre-diabet* OR glucose OR glycemic OR glycemia OR hyperglycemia OR glycated hemoglobin OR hypertensi* OR blood pressure OR cholesterol OR HbA1c).mp. AND (trial* OR randomized OR randomly OR rct* OR control group* OR clinical stud*).ti,ab. OR randomized controlled trial.pt Limit 2008 - ; English
Embase (OVID) 1988–	(money OR cash OR incentiv* OR token econom* OR token reinforcement* OR payment* OR paid OR earn* OR reimburse* OR wage* OR contingency management OR coupon* OR discount* OR voucher* OR gift* OR free food OR prize* OR award* OR reward*).mp. AND (Weight reduction OR weight management OR weight loss* OR obesity OR overweight OR body weight OR body mass OR bmi OR excess weight OR diet* OR feeding behavio?r OR eating OR nutrition OR lifestyle OR life style OR behavio?r change OR behavio?r modification OR behavio?r therapy OR health promotion OR health behavio?r OR healthy OR fitness OR exercise OR exercising OR physical activit* OR strength training OR diabetes OR diabetic OR prediabet* OR pre-diabet* OR glucose OR glycemic OR glycemia OR hyperglycemia OR glycated hemoglobin OR hypertensi* OR blood pressure OR cholesterol OR HbA1c).mp. AND (trial* OR randomi?ed OR randomly OR rct* OR control group* OR clinical stud*) Limit 2008 - ; English ; exclude Medline journals
PsycINFO (OVID) 1806–	(money OR cash OR incentiv* OR token econom* OR token reinforcement* OR payment* OR paid OR earn* OR reimburse* OR wage* OR contingency management OR coupon* OR discount* OR voucher* OR gift* OR free food OR prize* OR award* OR reward*).mp. AND (Weight reduction OR weight management OR weight loss* OR obesity OR overweight OR body weight OR body mass OR bmi OR excess weight OR diet* OR feeding behavio?r OR eating OR nutrition OR lifestyle OR life style OR behavio?r change OR behavio?r modification OR behavio?r therapy OR health promotion OR health behavio?r OR healthy OR fitness OR exercise OR exercising OR physical activit* OR strength training OR diabetes OR diabetic OR prediabet* OR pre-diabet* OR glucose OR glycemic OR glycemia OR hyperglycemia OR glycated hemoglobin OR hypertensi* OR blood pressure OR cholesterol OR HbA1c).mp. AND (trial* OR randomi?ed OR randomly OR rct* OR control group* OR clinical stud*) Limit 2008 - ; English
Cochrane Library	(money OR cash OR incentiv* OR “token econom*” OR “token reinforcement*” OR payment* OR paid OR earn* OR reimburse* OR wage* OR “contingency management” OR coupon* OR discount OR voucher* OR gift* OR “free food” OR prize* OR award* OR reward*):ti AND (“Weight reduction” OR “weight management” OR “weight loss*” OR obesity OR overweight OR “body weight” OR “body mass” OR bmi OR “excess weight” OR diet* OR “feeding behavio?r” OR eating OR nutrition OR lifestyle OR “life style” OR “behavio?r change” OR “behavio?r modification” OR “behavio?r therapy” OR “health promotion” OR “health behavio?r” OR healthy OR fitness OR exercise OR exercising OR “physical activit*” OR “strength training” OR diabetes OR diabetic OR prediabet* OR pre-diabet* OR glucose OR glycemic OR glycemia OR hyperglycemia OR “glycated hemoglobin” OR hypertensi* OR “blood pressure” OR cholesterol OR HbA1c):ti,ab Limit 2008 - ; English

^a^ Duplicates were identified using the EndNote (Clarivate) automated "find duplicates" function with preference set to match on title, author and year, and removed from your EndNote library. Additional duplicates will likely be found that EndNote was unable to detect.

## Post-Test Information

To obtain credit, you should first read the journal article. After reading the article, you should be able to answer the following, related, multiple-choice questions. To complete the questions (with a minimum 75% passing score) and earn continuing medical education (CME) credit, please go to http://www.medscape.org/journal/pcd. Credit cannot be obtained for tests completed on paper, although you may use the worksheet below to keep a record of your answers.

You must be a registered user on http://www.medscape.org. If you are not registered on http://www.medscape.org, please click on the “Register” link on the right hand side of the website.

Only one answer is correct for each question. Once you successfully answer all post-test questions, you will be able to view and/or print your certificate. For questions regarding this activity, contact the accredited provider, CME@medscape.net. For technical assistance, contact CME@medscape.net. American Medical Association’s Physician’s Recognition Award (AMA PRA) credits are accepted in the US as evidence of participation in CME activities. For further information on this award, please go to https://www.ama-assn.org. The AMA has determined that physicians not licensed in the US who participate in this CME activity are eligible for *AMA PRA Category 1 Credits™*. Through agreements that the AMA has made with agencies in some countries, AMA PRA credit may be acceptable as evidence of participation in CME activities. If you are not licensed in the US, please complete the questions online, print the AMA PRA CME credit certificate, and present it to your national medical association for review.

## Post-Test Questions

### Study Title: Effectiveness of Incentives for Improving Diabetes-Related Health Indicators in Chronic Disease Lifestyle Modification Programs: a Systematic Review and Meta-Analysis

#### CME Questions

1. What was the most common patient incentive employed in the 19 randomized trials included in the current meta-analysis?
  - Gift cards
  - Coupons
  - Cash
  - Free transportation
2. Which one of the following statements regarding the effect of patient incentives on body weight/body mass index (BMI) in the current study is most accurate?
  - Compared with the control group, the incentive group lost an average of 6 kg and experienced a decline in BMI of 2 mg/kg^2^
  - Compared with the control group, the incentive group lost an average of 2 kg and experienced a decline in BMI of 0.5 mg/kg^2^
  - Incentives had no effect on body weight or BMI
  - Participants in the incentive groups experienced a slight increase in body weight and BMI compared with the control group
3. How did patient incentives affect blood pressure values in the current study?
  - Incentives failed to improve either systolic or diastolic blood pressure
  - Incentives improved systolic, but not diastolic, blood pressure
  - Incentives improved diastolic, but not systolic, blood pressure
  - Incentives improved both systolic and diastolic blood pressure
4. Which one of the other following cardiometabolic variables was most improved with patient incentives in the current study?
  - Total cholesterol
  - Waist circumference
  - Average daily steps on a pedometer
  - HbA_1c_
